# In-Depth Analysis of the Antibody Response of Individuals Exposed to Primary Dengue Virus Infection

**DOI:** 10.1371/journal.pntd.0001188

**Published:** 2011-06-21

**Authors:** Ruklanthi de Alwis, Martina Beltramello, William B. Messer, Soila Sukupolvi-Petty, Wahala M. P. B. Wahala, Annette Kraus, Nicholas P. Olivarez, Quang Pham, James Brian, Wen-Yang Tsai, Wei-Kung Wang, Scott Halstead, Srisakul Kliks, Michael S. Diamond, Ralph Baric, Antonio Lanzavecchia, Federica Sallusto, Aravinda M. de Silva

**Affiliations:** 1 Department of Microbiology and Immunology, and the Southeast Regional Center of Excellence for Biodefense and Emerging Infectious Diseases Research, University of North Carolina School of Medicine, Chapel Hill, North Carolina, United States of America; 2 Institute for Research in Biomedicine, Bellinzona, Switzerland; 3 Departments of Medicine, Molecular Microbiology, Pathology and Immunology, and the Midwest Regional Center for Biodefense and Emerging Infectious Diseases Research, Washington University School of Medicine, St. Louis, Missouri, United States of America; 4 Department of Tropical Medicine, Medical Microbiology and Pharmacology, John A. Burns School of Medicine, University of Hawaii at Manoa, Honolulu, Hawaii, United States of America; 5 Pediatric Dengue Vaccine Initiative, International Vaccine Institute, Seoul, Korea; Texas Biomedical Research Institut, United States of America

## Abstract

Humans who experience a primary dengue virus (DENV) infection develop antibodies that preferentially neutralize the homologous serotype responsible for infection. Affected individuals also generate cross-reactive antibodies against heterologous DENV serotypes, which are non-neutralizing. Dengue cross-reactive, non-neutralizing antibodies can enhance infection of Fc receptor bearing cells and, potentially, exacerbate disease. The actual binding sites of human antibody on the DENV particle are not well defined. We characterized the specificity and neutralization potency of polyclonal serum antibodies and memory B-cell derived monoclonal antibodies (hMAbs) from 2 individuals exposed to primary DENV infections. Most DENV-specific hMAbs were serotype cross-reactive and weakly neutralizing. Moreover, many hMAbs bound to the viral pre-membrane protein and other sites on the virus that were not preserved when the viral envelope protein was produced as a soluble, recombinant antigen (rE protein). Nonetheless, by modifying the screening procedure to detect rare antibodies that bound to rE, we were able to isolate and map human antibodies that strongly neutralized the homologous serotype of DENV. Our MAbs results indicate that, in these two individuals exposed to primary DENV infections, a small fraction of the total antibody response was responsible for virus neutralization.

## Author Summary

Dengue is a mosquito-borne viral disease of humans. The dengue virus complex is made up of four viruses designated as serotypes. People experiencing their first infection develop immune responses that prevent re-infection with the same serotype only. People experiencing a second infection with a new serotype face a greater risk of developing a severe disease known as dengue hemorrhagic fever. Although studies indicate that antibodies can prevent or enhance disease caused by DENV, few studies have explored the specific properties of human antibodies against DENV. The objective of this study was to conduct a detailed analysis of the antibody response of two individuals who had recovered from primary infections. Human antibodies bound to sites on the dengue virus particle including the viral pre-membrane (prM/M) and envelope (E) proteins. Our studies indicate that the human antibody response consists of a minor population of strongly neutralizing antibody and a major population of DENV serotype cross-reactive, non-neutralizing antibody with potential for enhancement of virus and disease. Further studies with more DENV-immune subjects are needed to determine if our findings are broadly applicable to primary infections.

## Introduction

Dengue virus (DENV) complex consists of 4 serotypes. People exposed to primary DENV infections develop robust antibody responses that cross-react with all serotypes (Reviewed in [1]). Despite the extensive cross-reactivity, individuals only develop long term, protective immunity against the homologous serotype responsible for the primary infection [2], [3]. Indeed, the risk of progressing to DHF is greater during secondary compared to primary infection [4]. A prevailing theory that explains severe dengue during secondary infection is that pre-existing, non-neutralizing dengue specific antibodies enhance DENV entry and replication in Fc-receptor-bearing cells, which leads to a higher viremia and more severe disease [4]. Antibodies have been demonstrated to enhance DENV in cell culture [5], [6] and in animal models of dengue pathogenesis [7]–[9].

Our current understanding of how antibodies interact with DENV and other flaviviruses is primarily based on studies utilizing mouse monoclonal antibodies (MAbs) (Reviewed in [10]). The DENV envelope (E) protein is the principle target of neutralizing antibodies. Antibody neutralization occurs by blocking critical functions of the E protein, including attachment to host cells and low pH-dependent fusion of the viral and host cell membranes [11]. The crystal structures of the E protein of several flaviviruses have been solved [12]–[15]. Individual subunits of E protein consist of three beta-barrel domains designated domains I (EDI), II (EDII) and III (EDIII), with the native protein forming a head-to-tail homodimer. Mouse MAbs that bind to all three domains of DENV E have been generated and characterized [16]–[23]. Although neutralizing mouse MAbs have been mapped to all three domains of E, the most strongly neutralizing MAbs recognize epitopes on the lateral ridge and A strand of EDIII [24].

Following a primary DENV infection, humans develop antibodies that cross-react with all 4 serotypes, but mainly neutralize the homologous serotype responsible for the infection (Reviewed in[3]). Studies with human immune sera and, more recently, human monoclonal antibodies have demonstrated that the dominant antibody response is cross-reactive and weakly neutralizing [25]–[30]. Multiple viral antigens including E protein, pre-membrane (prM/M) protein and non-structural protein 1 (NSP1) are recognized by the human humoral response [25]–[30]. Nonetheless, few studies have defined the actual epitopes of DENV recognized by type-specific and cross-reactive human antibodies at the structural level and compared this to the epitopes defined using mouse antibodies. The target(s) of dengue type-specific, strongly neutralizing human antibodies remain unknown. The goal of this study was to study two subjects in-depth to define the major antigens and epitopes recognized by antibodies that develop following primary human DENV infection. Defining the human B-cell epitopes on DENV is a key step towards understanding how antibodies can both enhance and inhibit the severity of DENV infections.

## Materials and Methods

### Viruses, recombinant proteins and immune sera

DENV1 WestPac-74, DENV2 S-16803, DENV3 CH-53489, and DENV4 TVP-360, provided by Dr. Robert Putnak (Walter Reed Army Institute of Research, Silver Spring, MD) were used in the present study [29]. Recombinant envelope (rE) proteins from the 4 DENV serotypes were kindly provided by Dr. Beth-Ann Coller (Hawaii Biotech, Inc) [12]. The recombinant proteins bind to conformational MAbs and X-ray crystallography studies have demonstrated that these proteins retained a native-like structure [12], [13]. Convalescent DENV immune sera were obtained from volunteers who had experienced natural DENV infections during travel abroad. The protocol for recruiting and collecting blood samples from people was approved by the Institutional Review Board of the University of North Carolina at Chapel Hill. Written informed consent was obtained from all subjects before collecting blood.

### Whole DENV and recombinant E antigen ELISAs

ELISA plates were coated with 50 ng of purified virus or 100 ng of rE in carbonate buffer at pH 9.6 for 2 hrs at room temperature and incubated with blocking buffer (0.05% TBS-T containing 3% skim milk or 3% normal goat serum) at 37^o^C for 1 hr. Human immune sera or hMAbs serially diluted in blocking buffer were added for 1 hr at 37°C followed by alkaline phosphatase-conjugated goat anti-human IgG (Sigma) for 1 hr at 37°C. Finally, p-nitrophenyl phosphate substrate (Sigma) was added to each well and the reaction was allowed to develop for 15 minutes before recording optical density at 405 nm on a spectrophotometer.

### DENV Neutralization assays

DENV neutralizing antibodies was measured by a focus reduction neutralization test (FRNT) with Vero cells or using a flow cytometry-based neutralization assay with the U937 human monocytic cell line stably transfected with DC-SIGN as previously described [31].

### Production of human MAbs (hMAbs) from dengue immune travelers

Peripheral blood samples were obtained from two healthy adult donors who were infected by DENV during foreign travel. The dengue neutralization profiles confirmed previous primary DENV2 (Donor 013) and DENV3 (Donor 033) infections (Table S1). From both donor hMAbs were produced as previously described [32]. B-cells producing DENV specific antibody were identified by screening culture supernatants by flow cytometry for antibodies that bound to C6/36 insect cells infected with DENV2 (Donor 013) or DENV3 (Donor 033) [32]. A secondary screen to identify antibodies that bound to the rE was performed by ELISA as previously described [32].

### Epitope mapping of EDIII binding hMAbs

DENV antibody escape mutant viruses were selected for by infecting Vero cells with DENV2 (strain S-16803) in the presence of hMAb concentrations estimated to neutralize greater than 99% of infectious virus (i.e. 1.0 µg/ml for DVC 3.7 and 1.5 µg/ml for DVC 10.16). Equivalent GC copy numbers of control and antibody treated viruses were repeatedly passaged in the presence of hMAbs until equivalent DENV genomic copy numbers were observed for MAb treated and control samples (4–6 passages under antibody pressure). Escape mutant viruses were plaque purified and amplified. E genes were amplified by RT-PCR and sequenced to identify mutations linked to antibody escape. Antibody binding sites were also mapped by using yeast cells expressing a library of EDIII as previously described [33]. Mutations were mapped onto the DENV2 EDIII structure using the atomic coordinates of DENV2 EDIII (RCSB accession number 1OAN) and displayed using PyMOL Molecular Graphics System, Version 1.3 (Schrödinger, LLC).

## Results

The objective of the current study was to characterize the primary human antibody response to DENV by comparing immune sera and MAbs derived from two individuals previously exposed to primary DENV infections. Donor 033 had reported a high fever following a visit to India in 2005 and laboratory investigations confirmed a primary DENV3 infection (data not shown). One year later, when serum and peripheral blood mononuclear cells (PBMCs) were isolated, the subject had a neutralizing antibody response that primarily targeted DENV3 (Table S1). Donor 013 developed a fever, clinically diagnosed as dengue, while visiting a Pacific Island in 1996. Eight years later when serum and PBMCs were isolated for the current study, the subject had a neutralizing antibody profile consistent with a past primary DENV2 infection (Table S1).

Initially we characterized the binding properties of serum polyclonal antibodies in both subjects using purified DENVs and recombinant DENV E proteins from the 4 serotypes. We calculated endpoint titers (reciprocal of highest serum dilution that was positive in the assay) to estimate relative levels of antibody against whole virus and rE antigens (Table 1). The quantity of antibody binding to E was only 10 to 35% of the quantity that bound to whole virus suggesting that antibodies bound to sites on the virus that were not present on rE protein (Table 1). Thus, in both subjects the predominant antibodies binding to dengue virions were serotype cross-reactive and directed to epitopes on the virus and, to a lesser extent, to epitopes on rE protein.

**Table 1:** Relative levels of virus and rE protein binding antibody in immune sera.

Serum	Serotype	End point binding titer^a^		rE antibody relative to whole virus antibody (%)^b^
		Whole virus	rE protein	
Serum 013 (primary DENV2)	DENV1	3,579	358	10
	DENV2	8,906	1,536	17
	DENV3	3,579	1,242	35
	DENV4	3,141	485	15
				
Serum 033 (primary DENV3)	DENV1	13,164	1,338	10
	DENV2	8,906	2,050	23
	DENV3	20,768	6,931	33
	DENV4	12,334	1,815	15

a The end point binding titers are based on ELISAs performed with virus or recombinant E (rE) protein antigen and serial dilutions of immune serum. The end point titer was the reciprocal of the highest dilution that produced a signal 2 standard deviations above the signal for normal human sera.

b The amount of antibody that bound to rE protein relative to the virus was calculated using the following formula: (end point titer for rE antigen/end point titers for whole virus)X 100. When endpoint titers were calculated using a E protein cross reactive mouse MAb (4G2), similar end point titers were obtained for well coated with virus or recombinant E indicating both antigens had a similar number of accessible E molecules.

### Identification of DENV-reactive memory B cells following primary infection

We have previously reported that dengue reactive memory B cells are common following both primary and secondary DENV infection [30]. To further characterize the human B cell response in donor 033, we immortalized memory B cells. PBMCs were isolated and IgG+ memory B cells were immortalized with EBV and CpG as previously described [34]. The immortalized B cell culture supernatants were screened for antibodies that bound to C6/36 insect cells infected with DENV3. Thirty five percent of the B cell cultures generated from donor 033 were positive following this initial screen (Table 2). From the dengue positive cultures only 7.5% of the cultures bound to rE protein (Table 2). To further characterize the binding and functional properties of human antibodies, we isolated hMAbs from donor 033.

**Table 2:** Screen for isolating DENV-specific human Mabs.

Donor	No. of positive cultures after primary screen^a^	Proportion of DENV positive cultures binding to rE protein^b^	No. of human MAbs produced^c^	Reference
033 (primary DENV3)	332/960 (35%)	7.50%	16	This study
013 (primary DENV2)	567/2016 (28%)	2.90%	10	Betramello et al 2010

a Primary screen was conducted by flow cytometry using C6/36 cells infected with the homologous serotype.

b Proportion of recombinant E (rE) protein reactive cultures was determined by ELISA using recombinant protein from homologous virus.

c For donor 013, the selection of cultures for DENV-specific MAb production was biased to enrich for rE and EDIII reactive MAbs.

### Isolation and characterization of DENV-specific hMAbs from donor 033

**Figure 1:**
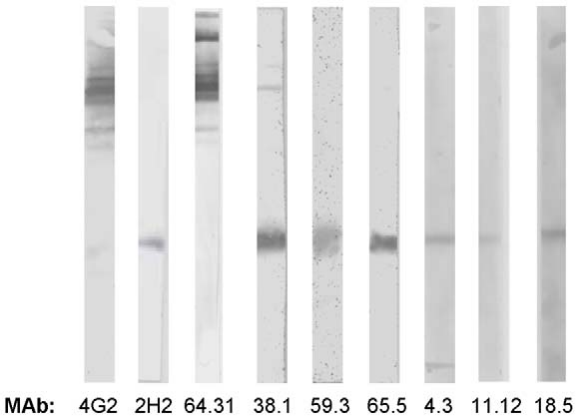
Antigens recognized by hMAbs produced from donor 033. DENV3 virions were purified and the viral proteins were separated by SDS-polyacrylamide gel electrophoresis. Western blots were performed to identify the viral antigens recognized by hMAbs from donor 033. The figure displays Western blot results for selected hMAbs that bound to E protein (64.31) and prM protein (DV38.1, 59.3, 65.5, 4.3, 11.12, and 18.5). 4G2 and 2H2 are control mouse MAbs that bind E and prM proteins, respectively.

To isolate hMAbs specific for DENV from donor 033, positive B cell cultures were cloned by limiting dilution and 16 clones were isolated and expanded. All the hybridomas produced IgG1 with the single exception DV20.10, which was IgG3 (Table S2). To identify antibodies that bound to structural viral antigens, the hMAbs were tested for binding to purified DENV3 particles in ELISA. Fourteen out of 16 hMAbs bound to DENV3 virus. Of the 14 hMAbs that bound to DENV3 particles, 13 cross-reacted with all four dengue serotypes dengue complex reactiveand one antibody (hMAb DV51.3) bound to serotypes 1 and 3, but not 2 and 4 (dengue sub complex reactive)(Table S2). Dengue virions were solubilized and subjected to Western blot analysis to identify the viral structural proteins recognized by hMAbs. Ten of the 14 hMAbs bound to prM and only a single hMAb (DV64.3) bound to E protein (Figure 1). Three hMAbs did not bind to viral antigens by Western blot, although they reacted with DENV3 virion. The 16 hMAbs were also tested for binding to rE protein in ELISA. Only hMAb DV64.31, which had also recognized E protein by Western Blot, bound to rE protein from all 4 DENV serotypes (Table S2).

**Figure 2:**
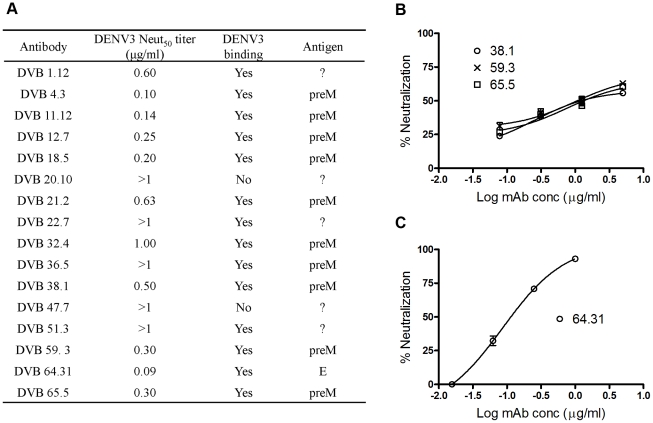
DENV3 neutralization by donor 033 human MAbs. The 50% neutralization titers against DENV3 were determined for all 16 hMAbs from donor 033 using a flow cytometry based neutralization test that utilizes U937 cells expressing DC-SIGN. **(A)** Dengue antigen recognized and the 50% neutralization titer for each hMAb. The hMAbs for which a specific antigen has still not been identified are designated with a *?*. **(B)** Neutralization curves for three representative prM antibodies. Note that prM antibodies have shallow neutralization curves, which plateau when ∼60% of the virions are neutralized. **(C)** Neutralization curve of the only E protein reactive antibody obtained from donor 033. This antibody has a steeper neutralization curve compared to prM antibodies.

We next tested the ability of the 16 antibodies generated from donor 033 to neutralize DENV. Five hMAbs including the two that did not bind to the virion lacked neutralizing activity (Figure 2). The remaining antibodies ranged from weak to moderately neutralizing and had DENV3 50% neutralization titers that ranged from 0.09 to 1 µg/ml (Figure 2A). The neutralizing antibodies had similar 50% neutralization titers against all 4 serotypes, which was consistent with their broad binding specificity (data not shown). In general, prM antibodies had neutralization curves that were shallow and did not reach 100% neutralization, indicating that a fraction of the virus population was resistant to antibody neutralization (Figure 2B). The single E reactive antibody (DV64.31) exhibited a steeper neutralization curve and neutralized 100% of virus at high concentrations (Figure 2C). In summary, the hMAbs generated from donor 033, who had experienced a primary DENV3 infection, were broadly cross-reactive, weakly neutralizing, and mainly directed to epitopes on prM. None of the hMAbs mimicked the functional properties of immune serum from donor 033 that displayed strong type-specific neutralization of DENV3 (Table S1). A complete summary of the functional profiles of all sixteen hMAbs from donor 033 is included as supplementary material (Table S2).

### Characterization of Human MAbs generated from donor 013

Next, we characterized the dengue specific memory B cell response in donor 013, who had recovered from a primary DENV2 infection. We have previously reported on some of the properties of B cells and hMAbs generated from this donor [30]. Here we expand on these previous results by comparing properties of serum antibodies and hMAbs from this donor and by epitope mapping a subset of hMAbs from this donor. When immortalized B cell culture supernatants from donor 013 were screened for antibodies that bound to C6/36 insect cells infected with DENV2, 28% (567/2016) of the cultures were found positive for DENV-specific B cells following this initial screen (Table 2). From the DENV positive cultures only 2.9% of the cultures bound to rE protein (Table 2). Thus, as in the case of donor 033, although dengue specific memory B cells were frequent, only a small fraction of the positive cultures from donor 013 produced antibodies that bound to rE protein.

**Figure 3:**
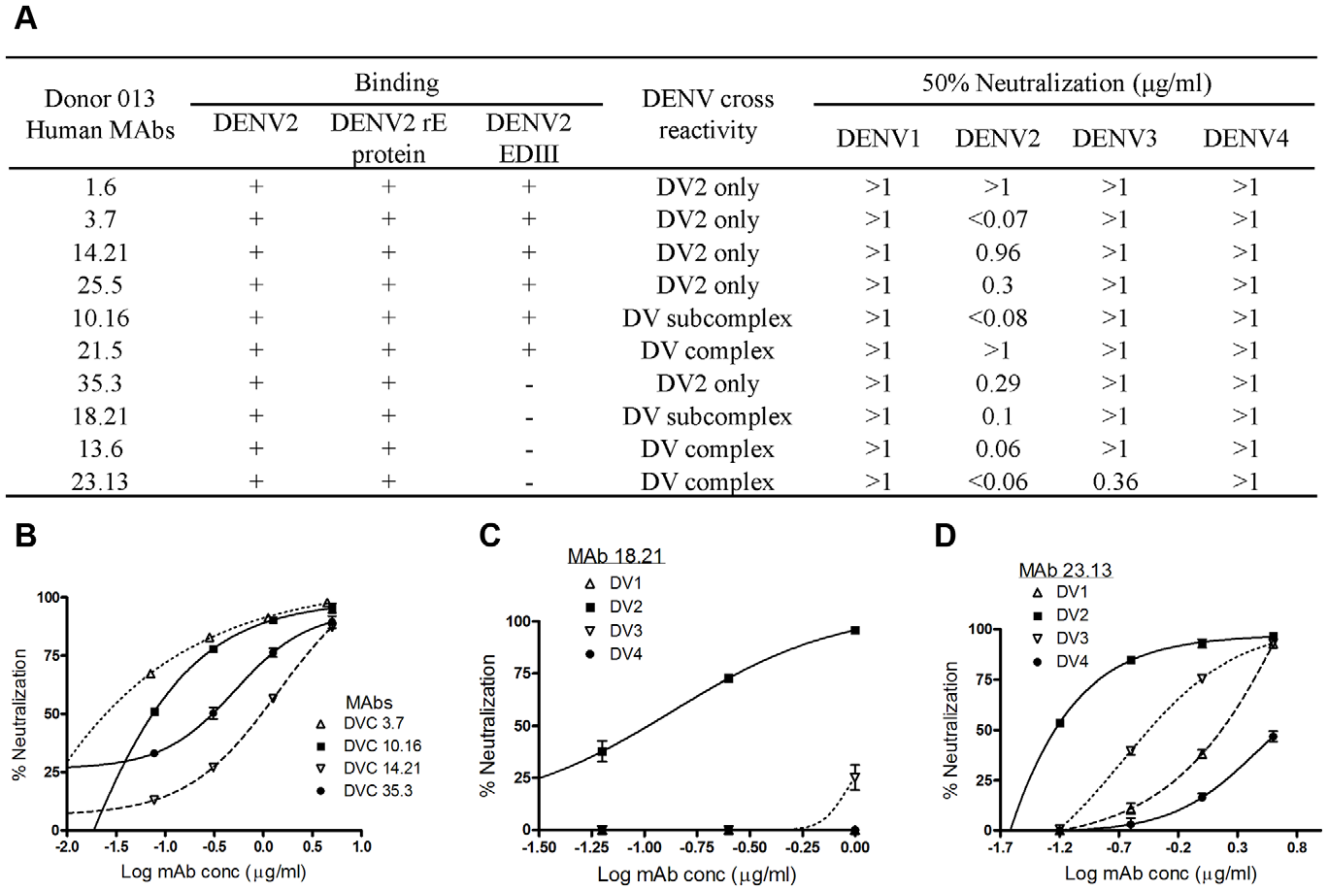
Binding and neutralization properties of donor 013 hMAbs. Ten hMAbs from donor 013 were tested for binding dengue virus, recombinant E (rE) and EDIII from DENV2. Cross-reactivity was determined by using whole virus antigen from all four serotypes. For each hMAb the 50% neutralization titer was determined using flow cytometry and U937 cell expressing DC-SIGN. **(A)** Summary of the binding and neutralization data for all hMAbs from donor 013. **B, C and D** display representative neutralization curves for DENV2 type-specific **(B)**, subcomplex- specific **(C)** and complex-specific **(D)** hMAbs.

Since a relatively unbiased selection scheme for producing hMAbs from donor 033 indicated that most hMAbs were cross-reactive, weakly neutralizing and directed to antigens other than rE, we altered the selection scheme for donor 013 to enrich for hMAbs that recognized rE from DENV2. After the initial screening of memory B cell culture supernatants, the relatively rare rE protein binding cultures were selected for cloning and expansion. Ten rE binding hMAbs were produced (all IgG1). When the antibodies were tested for binding to heterologous serotypes, 5 hMAbs were DENV2 type-specific, 2 hMAbs were dengue subcomplex-specific and 3 hMAbs were dengue complex-specific (Figure 3A). Six of the ten hMAbs bound to EDIII from DENV2 (Figure 3A). The hMAbs from donor 013 displayed variable neutralization properties (Figure 3A–C), with 2 non-neutralizing hMAbs (DV1.6, and 21.5; 50% neutralization titers >1 ug/ml) , 4 weakly to moderately neutralizing hMAbs (DV14.21, 35.3, 25.5 and 18.21; 50% neutralization titers between 0.1 and 1 ug/ml) and 4 strongly neutralizing hMAbs (DV3.7, 10.16, 13.6 and 23.13; 50% neutralization titers <0.1 ug/ml). The strongly neutralizing hMAbs, including those that were cross-reactive in binding assays, neutralized DENV2 better than the other serotypes (Figure 3). Thus, by biasing the initial screen for hMAbs that bound to rE protein, we identified hMAbs with neutralization profiles that were more similar to the immune sera of donor 013, which strongly neutralized DENV2.

### Epitope mapping donor 013 hMAbs binding to EDIII

**Figure 4:**
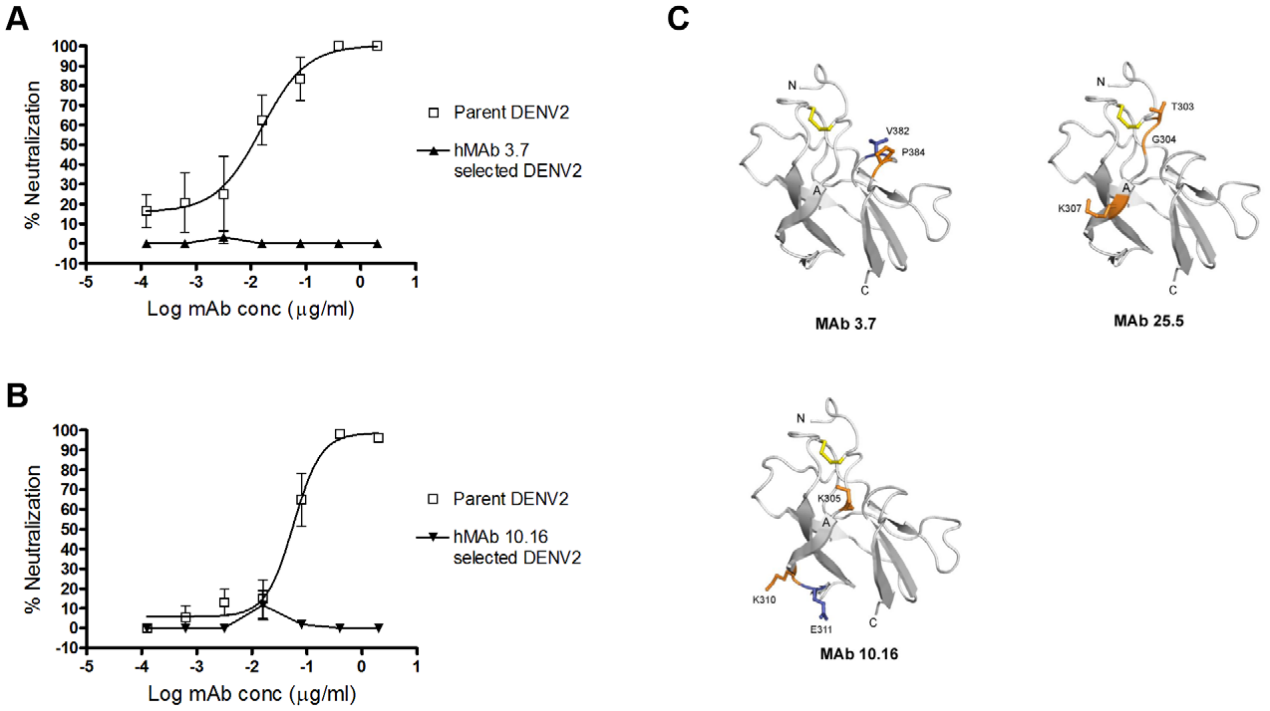
Epitope mapping of anti-DENV2 hMAbs binding to EDIII. To identify antibody binding sites, DENV2 was serially passaged in the presence of neutralizing hMAbs DV3.7 or DV10.16. Viruses growing in the presence of hMAbs were plaque purified and expanded. Neutralization escape was confirmed by growing the parental and the cloned antibody selected viruses in the presence of each hMAb 3.7 **(A)** or hMAb 10.16 **(B)**. **(C)** Localization of neutralizing human antibody epitopes on the structure of DENV2 EDIII (strain 16681) using residues identified by neutralization escape selection (*blue*) or yeast surface display screening (see Table 3) (*orange*). Ribbon diagram of DENV-2 EDIII was generated from a published X-ray crystallographic structure. The disulfide bond is highlighted in yellow. Human MAbs 3.7 and 25.5 are type-specific antibodies that bind to epitopes centered on the lateral ridge while 10.16 is sub-complex-specific and bind to an epitope centered on the A strand of EDIII.

Several hMAbs from donor 013 bound to EDIII (Figure 3). Two approaches were used to map the binding sites of these hMAbs on EDIII. Two strongly neutralizing hMAbs that bound EDIII, which were type- (DV3.7) or subcomplex- (DV10.16) specific, were mapped by identifying neutralization escape variants after passaging DENV2 in the presence of each antibody (ired a mutation on EDIII. The virus passaged in the presence of hMAb DV3.7 acquired the single point mutation V382G (Table 3 and Figure 4C). This residue is located on the EDIII lateral ridge, which is a target of previously mapped type-specific strongly neutralizing mouse MAbs [17], [23] (Table 3 and Figure 4C). The virus passaged in the presence of hMAb DV10.16 acquired the point mutation E311K (Table 3 and Figure 4C). This amino acid is located on the A strand of EDIII and forms part of a dengue subcomplex epitope recognized by neutralizing mouse MAbs [18], [23] (Table 3 and Figure 4C).

**Table 3:** Epitope mapping DENV2 EDIII reactive MAbs from donor 013.

Human MAb (Donor 013)^a^	Binding specificity^b^	DENV2 neutralization potency^c^	Neutralization escape mutations^d^	DENV2 Yeast EDIII mapping^e^		EDIII Epitope
				Loss of binding	Decreased binding	
DV3.7	DENV2 type specific	Strong	V382G	P384A, P384N	K307Q	DIII-lateral
DV14.21	DENV2 type specific	Weak	*NA*	K305E, K307N, K307I, K310E	G304Y K307Q	DIII-A strand
DV25.5	DENV2 type specific	Moderate	*NA*	T303Y, G304Y, K307N, K307Q	D329Q, G330D, E383G, P384A, P384N	DIII-lateral
DV10.16	DENV subcomplex	Strong	E311K	K305E, K310E	*NA*	DIII-A strand
DV21.5	DENV complex	None	*NA*	K305E, K310E, K317Y	*NA*	DIII-A strand

*NA  = * Not Applicable

a Only EDIII reactive MAbs from donor 013 were mapped.

b The MAbs were tested for binding to the four DENV serotypes.

c Neutralization potency is based on 50% neutralization titers in Figure 3.

d DENV2 escape mutants were obtained with strongly neutralizing MAbs only.

e EDIII mutations that led to a complete loss of binding and partial loss of binding are indicated separately.

As an independent approach to mapping EDIII-reactive hMAbs, we used a yeast surface display assay that has been previously used to map numerous flavivirus antibodies [22], [23], [33]. Table 3 summarizes all mutations that resulted in loss of binding or neutralization of EDIII antibodies generated from donor 013. All the human EDIII-binding MAbs mapped to the lateral ridge or the A strand regions that have previously been described as targets of mouse MAbs [23] (Figure 4C).

## Discussion

Our main objective was to define viral antigens and epitopes recognized by 2 individuals exposed to primary DENV infections. In both these subjects the dengue virion was a major target of the humoral immune response but many of these antibodies did not bind to rE protein. The DENV virion displays 180 E and 180 prM or M proteins that are arrayed with pseudo-icosahedral symmetry. Depending on the maturation state of the virus particle, the 180 E protein molecules are organized as 90 head-to-tail dimers that lie flat on the virion surface or 60 trimers that protrude as spikes from the surface [35]. Since the E protein, with ∼500 amino acids, is considerably larger than the 166 amino acid prM protein, the majority of surface exposed viral protein consists of the ectodomain of E. Thus, our finding that many antibodies bound to epitopes on the virus that were not preserved on rE was somewhat unexpected. These findings are consistent with the results of another study from our group [30] as well as a study by Dejnirattisai and colleagues who observed that many hMAbs that bound to DENV particles did not bind to rE protein [25]. As the rE protein used in the current study lacked ∼20% of the protein, which include the membrane proximal regions and the transmembrane domains, it is possible that some antibodies bind to these regions. Moreover, antibodies may recognize E protein epitopes that are only available in the context of the native oligomeric array on the virion. Several hMAbs generated from individuals infected with West Nile virus bound the virion but not rE protein; these hMAbs recognized epitopes that were created by adjacent E protein molecules on the surface of the virion or formed by hinge regions [36].

When producing MAbs from donor 013, we increased the probability of identifying strongly neutralizing antibodies by biasing the selection scheme for MAbs that specifically recognized rE protein. In contrast to the weak or non-neutralizing hMAbs isolated from donor 033, several hMAbs from donor 013 displayed strong type-specific DENV2 neutralization. Of note, even hMAbs from donor 013 that bound to more than one serotype (dengue subcomplex or complex MAbs) displayed type-specific neutralization of DENV2, which was the serotype that infected donor 013. Thus, by biasing the selection to enrich for rare rE protein binding antibodies, we generated hMAbs from donor 013 that were functionally similar to the polyclonal immune serum from the same donor that neutralized only DENV2. It is unclear how the results with hMAbs relate to the neutralization properties of polyclonal immune serum from these donors. One possibility is that the abundance of hMABs reflects the functional properties of antibodies in immune sera, where a small fraction of DENV-specific antibodies in immune sera are responsible for neutralization. Alternatively, it is conceivable that individual antibodies that are weakly neutralizing become strongly neutralizing due to cooperative effects that only occur in a polyclonal milieu. Further studies are needed to understand how the properties of hMABs from DENV immune donors relate to the properties of circulating serum antibody.

From the neutralizing MAbs from donor 013 that bound to rE, some bound to the lateral ridge and A strand epitopes on EDIII (ref) . We also identified several neutralizing hMABs (35.3, 18.21, 13.6, 23.13) that bound to rE but not EDIII and these antibodies most likely bind to epitopes on EDI or EDII. We are especially interested in mapping the binding sites of these hMABs as recent studies indicate that epitopes other than the lateral ridge and A strand of EDIII can be the targets of strongly neutralizing mouse, horse and primate antibodies [29], [37], [38], [7], [22],

Our results indicating that prM was a dominant target of the primary antibody response are in agreement with other recent studies [25], [30]. The prM protein is required for the proper folding and assembly of flavivirus particles in the endoplasmic reticulum [39], while also preventing adventitious fusion of the virus [40] in the acidic environment of the trans-Golgi network. Recent studies have demonstrated that DENV virions produced in cell culture are often partially processed and contain a mixture of unprocessed prM and fully processed M [41]. The maturation state and relative amount of prM on a virion can alter the potency of antibodies that bind specific epitopes on E, including the cross-reactive antibodies that bind the fusion loop in DII [42]. Additionally, immature, non-infectious particles became infectious in the presence of prM antibody by enhancing the ability of immature dengue virions to infect Fc-receptor bearing cells in vitro [25], [43]. Given our findings and those of others [25] establishing that prM antibodies are common following primary and secondary DENV infections, more work needs to be performed to address their contribution to dengue pathogenesis in humans.

In summary, the studies reported here demonstrate an unexpected antibody profile in two individuals following primary dengue infection. In both individuals a majority of the DENV-specific human antibodies were broadly cross-reactive and weakly neutralizing. Many antibodies bound to prM and sites on the virus that were not preserved on rE protein. Only a minor fraction of the total dengue specific antibody response was responsible for potent neutralization of the homologous virus. Given the difficulty of identifying suitable donors and generating antigen specific hMAbs, we only characterized the antibody response in two subjects here. Further studies with more dengue immune subjects are needed to determine if our findings are broadly applicable to primary dengue exposure.

## Supporting Information

Table S1 **Dengue immune human sera used in the present study.**
(DOC)

Table S2 **Properties of MAbs from donor 033 (Primary DENV3 infection).**
(DOC)

## References

[1] Roehrig JT (2003). Antigenic structure of flavivirus proteins.. Adv Virus Res.

[2] Halstead SB (2002). Dengue.. Curr Opin Infect Dis.

[3] Rothman AL (2004). Dengue: defining protective versus pathologic immunity.. J Clin Invest.

[4] Halstead SB (2003). Neutralization and antibody-dependent enhancement of dengue viruses.. Adv Virus Res.

[5] Halstead SB, Marchette NJ, Sung Chow JS, Lolekha S (1976). Dengue virus replication enhancement in peripheral blood leukocytes from immune human beings.. Proc Soc Exp Biol Med.

[6] Halstead SB, Chow JS, Marchette NJ (1973). Immunological enhancement of dengue virus replication.. Nat New Biol.

[7] Goncalvez AP, Engle RE, St Claire M, Purcell RH, Lai CJ (2007). Monoclonal antibody-mediated enhancement of dengue virus infection in vitro and in vivo and strategies for prevention.. Proc Natl Acad Sci U S A.

[8] Balsitis SJ, Williams KL, Lachica R, Flores D, Kyle JL (2010). Lethal antibody enhancement of dengue disease in mice is prevented by Fc modification.. PLoS Pathog.

[9] Zellweger RM, Prestwood TR, Shresta S (2010). Enhanced infection of liver sinusoidal endothelial cells in a mouse model of antibody-induced severe dengue disease.. Cell Host Microbe.

[10] Pierson TC, Diamond MS (2008). Molecular mechanisms of antibody-mediated neutralisation of flavivirus infection.. Expert Rev Mol Med.

[11] Pierson TC, Fremont DH, Kuhn RJ, Diamond MS (2008). Structural insights into the mechanisms of antibody-mediated neutralization of flavivirus infection: implications for vaccine development.. Cell Host Microbe.

[12] Modis Y, Ogata S, Clements D, Harrison SC (2003). A ligand-binding pocket in the dengue virus envelope glycoprotein.. Proc Natl Acad Sci U S A.

[13] Modis Y, Ogata S, Clements D, Harrison SC (2005). Variable surface epitopes in the crystal structure of dengue virus type 3 envelope glycoprotein.. J Virol.

[14] Nybakken GE, Nelson CA, Chen BR, Diamond MS, Fremont DH (2006). Crystal structure of the West Nile virus envelope glycoprotein.. J Virol.

[15] Rey FA, Heinz FX, Mandl C, Kunz C, Harrison SC (1995). The envelope glycoprotein from tick-borne encephalitis virus at 2 A resolution.. Nature.

[16] Crill WD, Roehrig JT (2001). Monoclonal Antibodies That Bind to Domain III of Dengue Virus E Glycoprotein Are the Most Efficient Blockers of Virus Adsorption to Vero Cells. J Virol.

[17] Gromowski GD, Barrett AD (2007). Characterization of an antigenic site that contains a dominant, type-specific neutralization determinant on the envelope protein domain III (ED3) of dengue 2 virus.. Virology.

[18] Gromowski GD, Barrett ND, Barrett AD (2008). Characterization of dengue virus complex-specific neutralizing epitopes on envelope protein domain III of dengue 2 virus.. J Virol.

[19] Lin B, Parrish CR, Murray JM, Wright PJ (1994). Localization of a Neutralizing Epitope on the Envelope Protein of Dengue Virus Type 2.. Virology.

[20] Lok SM, Kostyuchenko V, Nybakken GE, Holdaway HA, Battisti AJ (2008). Binding of a neutralizing antibody to dengue virus alters the arrangement of surface glycoproteins.. Nat Struct Mol Biol.

[21] Roehrig JT, Bolin RA, Kelly RG (1998). Monoclonal Antibody Mapping of the Envelope Glycoprotein of the Dengue 2 Virus, Jamaica.. Virology.

[22] Sukupolvi-Petty S, Austin SK, Engle M, Brien JD, Dowd KA (2010). Structure and Function Analysis of Therapeutic Monoclonal Antibodies against Dengue Virus Type 2..

[23] Sukupolvi-Petty S, Austin SK, Purtha WE, Oliphant T, Nybakken GE (2007). Type- and Subcomplex-Specific Neutralizing Antibodies against Domain III of Dengue Virus Type 2 Envelope Protein Recognize Adjacent Epitopes. J Virol.

[24] Shrestha B, Brien JD, Sukupolvi-Petty S, Austin SK, Edeling MA (2010). The development of therapeutic antibodies that neutralize homologous and heterologous genotypes of dengue virus type 1.. PLoS Pathog.

[25] Dejnirattisai W, Jumnainsong A, Onsirisakul N, Fitton P, Vasanawathana S (2010). Cross-reacting antibodies enhance dengue virus infection in humans.. Science.

[26] Schieffelin JS, Costin JM, Nicholson CO, Orgeron NM, Fontaine KA (2010). Neutralizing and non-neutralizing monoclonal antibodies against dengue virus E protein derived from a naturally infected patient.. Virol J.

[27] Crill WD, Hughes HR, Delorey MJ, Chang GJ (2009). Humoral immune responses of dengue fever patients using epitope-specific serotype-2 virus-like particle antigens.. PLoS ONE.

[28] Lai CY, Tsai WY, Lin SR, Kao CL, Hu HP (2008). Antibodies to envelope glycoprotein of dengue virus during the natural course of infection are predominantly cross-reactive and recognize epitopes containing highly conserved residues at the fusion loop of domain II.. J Virol.

[29] Wahala WM, Kraus AA, Haymore LB, Accavitti-Loper MA, de Silva AM (2009). Dengue virus neutralization by human immune sera: role of envelope protein domain III-reactive antibody.. Virology.

[30] Beltramello M, Williams KL, Simmons CP, Macagno A, Simonelli L (2010). The human immune response to dengue virus is dominated by highly cross-reactive antibodies endowed with neutralizing and enhancing activity.. Cell Host Microbe.

[31] Kraus AA, Messer W, Haymore LB, de Silva AM (2007). Comparison of Plaque- and Flow Cytometry- Based Methods for Measuring Dengue Virus Neutralization.. J Clin Microbiol.

[32] Beltramello M, Williams KL, Simmons CP, Macagno A, Simonelli L (2010). The human immune response to dengue virus is dominated by highly cross-reactive antibodies endowed with neutralizing and enhancing activity.. Cell Host Microbe.

[33] Oliphant T, Nybakken GE, Engle M, Xu Q, Nelson CA (2006). Antibody Recognition and Neutralization Determinants on Domains I and II of West Nile Virus Envelope Protein 10.1128/JVI.01732-06.. J Virol.

[34] Traggiai E, Becker S, Subbarao K, Kolesnikova L, Uematsu Y (2004). An efficient method to make human monoclonal antibodies from memory B cells: potent neutralization of SARS coronavirus.. Nat Med.

[35] Mukhopadhyay S, Kuhn RJ, Rossmann MG (2005). A structural perspective of the flavivirus life cycle.. Nat Rev Microbiol.

[36] Vogt MR, Moesker B, Goudsmit J, Jongeneelen M, Austin SK (2009). Human monoclonal antibodies against West Nile virus induced by natural infection neutralize at a postattachment step.. J Virol.

[37] Sanchez MD, Pierson TC, Degrace MM, Mattei LM, Hanna SL (2007). The neutralizing antibody response against West Nile virus in naturally infected horses.. Virology.

[38] Oliphant T, Nybakken GE, Austin SK, Xu Q, Bramson J (2007). Induction of Epitope-Specific Neutralizing Antibodies against West Nile Virus 10.1128/JVI.00643-07.. J Virol.

[39] Yu IM, Zhang W, Holdaway HA, Li L, Kostyuchenko VA (2008). Structure of the immature dengue virus at low pH primes proteolytic maturation.. Science.

[40] Yu IM, Holdaway HA, Chipman PR, Kuhn RJ, Rossmann MG (2009). Association of the pr peptides with dengue virus at acidic pH blocks membrane fusion.. J Virol.

[41] Cherrier MV, Kaufmann B, Nybakken GE, Lok SM, Warren JT (2009). Structural basis for the preferential recognition of immature flaviviruses by a fusion-loop antibody.. Embo J.

[42] Nelson S, Jost CA, Xu Q, Ess J, Martin JE (2008). Maturation of West Nile virus modulates sensitivity to antibody-mediated neutralization.. PLoS Pathog.

[43] Rodenhuis-Zybert IA, van der Schaar HM, da Silva Voorham JM, van der Ende-Metselaar H, Lei HY (2010). Immature dengue virus: a veiled pathogen?. PLoS Pathog.

