# Correction: In-Depth Analysis of the Antibody Response of Individuals Exposed to Primary Dengue Virus Infection

**DOI:** 10.1371/annotation/f585335f-ff77-40ae-a8b6-ad6019af31aa

**Published:** 2011-08-12

**Authors:** Ruklanthi de Alwis, Martina Beltramello, William B. Messer, Soila Sukupolvi-Petty, Wahala M. P. B. Wahala, Annette Kraus, Nicholas P. Olivarez, Quang Pham, James Brian, Wen-Yang Tsai, Wei-Kung Wang, Scott Halstead, Srisakul Kliks, Michael S. Diamond, Ralph Baric, Antonio Lanzavecchia, Federica Sallusto, Aravinda M. de Silva

The ninth author's name was spelled incorrectly. The correct name is: James D. Brien.

The correct author contributions are: Conceived and designed the experiments: RdA MB WBM S-SP WMPBW AK NPO QP JDB W-YT W-KW SH SK MSD RB AL FS AMdS. Performed the experiments: RdA MB WBM S-SP WMPBW AK NPO QP JDB W-YT W-KW. Analyzed the data: RdA MB WBM S-SP WMPBW AK NPO QP JDB W-YT W-KW SH SK MSD RB AL FS AMdS. Contributed reagents/materials/analysis tools: AK MSD RB AL FS. Wrote the paper: RdA MB QP SH SK MSD RB AL FS AMdS.

