# Supplementary material for: In-Depth Analysis of the Antibody Response of Individuals Exposed to Primary Dengue Virus Infection
Source: PLoS Negl Trop Dis. 2011 Jun 21;5(6):e1188. doi: 10.1371/journal.pntd.0001188 (PMC3119640; doi:10.1371/journal.pntd.0001188)
Supplement: Table S1 — Dengue immune human sera used in the present study. (DOC) [file pntd.0001188.s001.doc]

| Table S1. Dengue immune human sera used in the present study | | | | | | | |
| --- | --- | --- | --- | --- | --- | --- | --- |
| Donor ID | Location & year of infection | Interval between infection and sample collection | PRNT50 titer*^a^* | | | | Infecting Serotype |
|  |  |  | DEN 1 | DEN 2 | DEN 3 | DEN 4 |  |
| 033*^b^* | India 2005 | 12 months | 1:49 | 1:129 | **> 1:1,280** | 1:119 | Primary DENV3 |
| 013 | South Pacific Island 1997 | 8 years | 1:178 | **> 1:1280** | 1:65 | 1:140 | Primary DENV2 |

*^a^* The 50 % plaque reduction neutralization titer was determined using Vero cells.

*^b^* DENV3 was isolated from the subject in 2005.
