# Supplementary material for: In-Depth Analysis of the Antibody Response of Individuals Exposed to Primary Dengue Virus Infection
Source: PLoS Negl Trop Dis. 2011 Jun 21;5(6):e1188. doi: 10.1371/journal.pntd.0001188 (PMC3119640; doi:10.1371/journal.pntd.0001188)
Supplement: Table S2 — Properties of MAbs from donor 033 (Primary DENV3 infection). (DOC) [file pntd.0001188.s002.doc]

| Table S2. Properties of MAbs from donor 033 (Primary DENV3 infection). | | | | | | | | | | | | |
| --- | --- | --- | --- | --- | --- | --- | --- | --- | --- | --- | --- | --- |
| MAb ID | IgG Isotype | Whole virus binding ELISA (OD_405_) | | | | Recombinant E protein ELISA (OD_405_) | | | | Recombinant DENV3 EDIII ELISA (OD 405) ^3^ | Target antigen (DENV3)^4^ | Neut_50_ (μg/ml) of DENV3 in U937 DC-SIGN cells |
|  |  | DENV1 | DENV2 | DENV3 | DENV4 | DENV1 | DENV2 | DENV3 | DENV4 |  |  |  |
| 1.12 | γ 1 | 0.48 | 0.76 | 1.13 | 1.04 | 0.06 | 0.07 | 0.06 | 0.07 | 0.00 | ? | 0.60 |
| 4.30 | γ 1 | 0.99 | 1.48 | 1.86 | 1.88 | 0.07 | 0.07 | 0.07 | 0.07 | 0.00 | preM | 0.10 |
| 11.12 | γ 1 | 1.08 | 1.77 | 1.78 | 1.95 | 0.07 | 0.07 | 0.06 | 0.06 | 0.00 | preM | 0.14 |
| 12.70 | γ 1 | 1.01 | 1.76 | 1.71 | 1.53 | 0.07 | 0.07 | 0.07 | 0.06 | 0.00 | preM | 0.25 |
| 18.50 | γ 1 | 1.18 | 1.69 | 1.72 | 1.86 | 0.07 | 0.10 | 0.08 | 0.09 | 0.00 | preM | 0.20 |
| 20.10 | γ 3 | 0.08 | 0.09 | 0.08 | 0.08 | 0.09 | 0.07 | 0.07 | 0.07 | 0.00 | ? | >1.00 |
| 21.20 | γ 1 | 1.17 | 1.68 | 1.63 | 1.63 | 0.07 | 0.07 | 0.07 | 0.06 | 0.00 | preM | 0.63 |
| 22.70 | γ 1 | 0.74 | 1.49 | 1.46 | 1.55 | 0.06 | 0.07 | 0.06 | 0.06 | 0.00 | ? | >1.00 |
| 32.40 | γ 1 | 1.01 | 1.68 | 1.63 | 1.59 | 0.06 | 0.07 | 0.06 | 0.06 | 0.00 | preM | 1.00 |
| 36.50 | γ 1 | 0.85 | 1.59 | 1.49 | 1.60 | 0.07 | 0.07 | 0.06 | 0.06 | 0.00 | preM | >1.00 |
| 38.10 | γ 1 | 1.09 | 1.65 | 1.62 | 1.62 | 0.06 | 0.07 | 0.07 | 0.07 | 0.00 | preM | 0.50 |
| 47.70 | γ 1 | 0.07 | 0.09 | 0.08 | 0.08 | 0.07 | 0.07 | 0.06 | 0.06 | 0.00 | ? | >1.00 |
| 51.30 | γ 1 | 2.16 | 0.09 | 0.82 | 0.09 | 0.07 | 0.07 | 0.06 | 0.06 | 0.00 | ? | >1.00 |
| 59. 3 | γ 1 | 1.27 | 1.93 | 1.72 | 1.59 | 0.06 | 0.07 | 0.07 | 0.07 | 0.00 | preM | 0.30 |
| 64.31 | γ 1 | 1.33 | 1.83 | 2.30 | 1.85 | 0.86 | 1.36 | 2.05 | 1.59 | 0.00 | E | 0.09 |
| 65.50 | γ 1 | 0.78 | 1.49 | 1.53 | 1.48 | 0.06 | 0.07 | 0.06 | 0.06 | 0.00 | preM | 0.30 |
